# Evaluation of Subjective Sleep Disturbances in Cancer Patients: A Cross-Sectional Study in a Radiotherapy Department

**DOI:** 10.3389/fpsyt.2021.648896

**Published:** 2021-03-18

**Authors:** Jun Wang, Bei-Yun Zhou, Chen-Lu Lian, Ping Zhou, Hui-Juan Lin, San-Gang Wu

**Affiliations:** Department of Radiation Oncology, The First Affiliated Hospital of Xiamen University, Xiamen, China

**Keywords:** cancer, radiotherapy, sleep disturbance, Pittsburgh sleep quality index, BMI, surgery

## Abstract

**Background:** The factors associated with sleep disturbances in cancer patients remains unclear. This study aimed to explore the prevalence of sleep disorders and predictors associated with sleep disturbance in cancer patients from a radiotherapy department.

**Methods:** Patients with cancers were recruited before the start of radiotherapy from our institution between January 2019 and February 2020. Pittsburgh Sleep Quality Index (PSQI) scale was used to assess sleep quality. Descriptive statistics, Chi-square test, and multivariate logistic regression analysis were used to conduct statistical analysis.

**Results:** A total of 330 eligible patients were included. Of them, 38.3% (*n* = 127) had the globe PSQI score >7, indicating that they suffered from sleep disorders. Patients with lung cancer (45.2%) were more likely to suffer from sleep disturbance, followed by cervical cancer (43.8%), nasopharyngeal carcinoma (41.7%), esophageal cancer (41.5%), breast cancer (37.7%), and colorectal cancer (30%). With regard to the PSQI components, the mean sleep duration was 8 h, 20.3% (*n* = 67) of them reported poor subjective sleep quality, 6.1% (*n* = 20) needed medication to improve sleep, and 53.6% (*n* = 177) suffered daytime dysfunction. Multivariate logistic regression models showed body mass index (BMI) ≥ 20 kg/m^2^ [odds ratio (OR) 0.599, 95% confidence interval (CI) 0.329–0.948, *P* = 0.031] and the receipt of surgery (OR 0.507, 95% CI 0.258–0.996, *P* = 0.048) were the significant favorable predictors for sleep disturbance, while age, gender, marital status, education level, comorbidity, metastasis status, diagnostic status, and cancer type were not significantly associated with sleep disturbance.

**Conclusions:** Approximately 40% of the cancer patients suffer from sleep disturbance before the start of radiotherapy. Patients with BMI ≥ 20 kg/m^2^ and receiving surgery are less likely to develop sleep disturbance in comparison with others.

## Introduction

Cancer is a major public health problem worldwide. Nowadays, the incidence of the tumor remains an increase over the past decades, and ~4,300,000 new cases and 2,800,000 cancer deaths occurred in 2015 in China (1). With the improvements of examinations and treatments, the mortalities associated with cancer have significantly declined in recent years (2, 3). Quality of life (QOL), such as sleep quality, social and physical function, and mental and emotional well-being, has attracted more attention from doctors in cancer-related departments (4).

Sleep disturbance, which is divided into the following categories: insomnia, hypersomnia, dyssomnia, day time sleepiness, circadian rhythm sleep-wake disturbance, sleep apnea, etc., seriously affects the physical and physiological health in the general population and cancer patients (5–9). The overall incidence of sleep disturbance in cancer patients ranged from 30 to 93.1%, which was significantly higher than that in the general population (9–33%) (5–9). Previous studies showed that poor sleep quality and shorter sleep duration were significantly associated with higher all-cause mortality in the general population (10, 11). Persistent poor sleep quality negatively affected the health condition in patients with cancers, including decreased psychological and physical function, poor sleep efficiency, and sleep duration were closely related to poor overall survival and cancer-specific death after a follow-up period of 10 years (12, 13). Therefore, it is important to explore the details of sleep status in the management of cancer patients.

The factors associated with sleep disturbances in cancer patients remain unclear. Previous studies have elaborated that age, higher body mass index (BMI), cancer-related fatigue, depression, and anxiety had a significant association with poor sleep quality among cancer patients (14, 15). However, most of the studies focused on the sleep quality of patients before the start or after the end of chemotherapy at present, and studies regarding radiotherapy were less elaborated. A study with 560 patients from the Netherlands showed that highly prevalent poor sleep quality was found among head and neck cancer patients before radiotherapy, however, it was not representative for other tumor types (16). Therefore, our study aimed to conduct a cross-sectional study to explore the prevalence of sleep disturbances and the details of sleep quality in cancer patients and to further identify the predictive factors associated with sleep disturbance before the start of radiotherapy.

## Materials and Methods

### Patients

Patients were recruited from the Department of Radiation Oncology, the First Affiliated Hospital of Xiamen University in China between January 2019 and February 2020. Patients who met the following criteria were eligible for inclusion in this study: (1) pathologically diagnosed with solid cancer; (2) aged more than 18 years old; (3) first hospitalized in our department; (4) not receiving preoperative radiotherapy; (5) having the ability of reading, writing, and understanding Chinese; (6) obtained written informed consent. Patients who have previously received radiotherapy, diagnosed with sleep disturbance (e.g., insomnia, hypersomnia, day time sleepiness, sleep apnea, etc.), refused to disclose sleep information and unable to finish the Pittsburgh Sleep Quality Index (PSQI) scale were excluded. The following sociodemographic variables were identified: age (<40, 40–60, >60 years), gender (male, female), marital status (married, unmarried, divorced), an education level (primary, middle school, high school, college, and above), comorbidity (comorbidity was defined as the simultaneous presence of cancer and other diseases, such as hypertension, diabetes, hepatitis B, etc.) (yes, no), BMI (<20 kg/m^2^, ≥ 20 kg/m^2^), distant metastasis (yes, no), surgery (yes, no), new diagnosis (new diagnosed patients were defined as patients who had diagnosed with cancer and had not received any treatment for their disease) (yes, no), and cancer type (head and neck cancer, thoracic tumor, breast cancer, abdominal tumor, other cancers). This study was approved by the ethics committee of the First Affiliated Hospital of Xiamen University.

### Instruments

The PSQI scale was devised by Buysse et al. to assess subjective sleep quality within the past month interval, which consisted of 19 self-rated items that generated seven component scores: subjective sleep quality (0–3), sleep latency (0–3), sleep duration (0–3), habitual sleep efficiency (0–3), sleep disturbances (0–3), use of sleep medication (0–3), and day time dysfunction (0–3). The global sleep quality score was summed by the above-mentioned seven component scores, and a total score of more than five was described as sleep impairment (range: 0–21) (17). However, previous validation studies of the Chinese version PSQI scale in cancer patients demonstrated that a cut-off value of seven showed better sensitivity and specificity (18, 19). Therefore, a PSQI score >7 was considered as sleep disturbance in this study.

### Statistical Analysis

Chi-square test was applied to assess possible differences of sociodemographic variables between the groups of sleep disturbance and normal sleep quality. Descriptive statistics (frequency distributions) were used to analyze the seven sleep component scores, and the PSQI scores were calculated according to established procedures. Multivariate logistic regression analysis was used to assess the independent predictive factors associated with sleep disturbance. IBM SPSS version 22.0 was used to conduct all the statistical analyses in this study, and a *P* < 0.05 (two-tails) was considered as statistically significant.

## Results

### Patient Characteristics

Table 1 showed the detailed information of patient characteristics. A total of 330 eligible patients were included in this study. Of them, 53% (*n* = 175) and 47% (*n* = 155) were male and female, respectively. A total of 13.9 (*n* = 46), 51.6 (*n* = 170), and 34.5% (*n* = 114) of the patients were <40, 40–60, and >60 years, respectively (median age: 54.5 years, range: 19–92 years). Approximately half of the patients (49.4%) received surgery. Most of the patients were married (88.5%), middle school or below (82.1%), without comorbidity (77.9%), BMI ≥ 20 kg/m^2^ (74.5%), and non-metastasis tumor (86.4%). With regard to specific tumor types, 26.1 (*n* = 86), 29.1 (*n* = 96), 16 (*n* = 53), 19.1 (*n* = 63), and 9.7% (*n* = 32) of them had head and neck cancer, thoracic tumor, breast cancer, abdominal tumor, and other cancer, respectively.

**Table 1:** Sociodemographic and clinical characteristics of the patients with and without sleep disturbance.

**Variables**	**Total (%)**	**Sleep disturbance**		***P***
		**No (%)**	**Yes (%)**	
**Gender**				
Male	175 (53)	112 (55.2)	63 (49.6)	0.324
Female	155 (47)	91 (44.8)	64 (50.4)	
**Age (years)**				
<40	46 (13.9)	32 (15.8)	14 (11)	0.475
40–60	170 (51.6)	103 (50.7)	67 (52.8)	
>60	114 (34.5)	68 (33.5)	46 (36.2)	
**Marital status**				
Married	292 (88.5)	177 (87.6)	115 (90.6)	0.413
Unmarried or divorced	38 (11.5)	25 (12.4)	12 (9.4)	
**Education level**				
Middle school or below	271 (82.1)	169 (83.7)	102 (80.3)	0.438
High school or above	59 (17.9)	33 (16.3)	25 (19.7)	
**Comorbidity**				
No	257 (77.9)	162 (79.8)	95 (74.8)	0.287
Yes	73 (22.1)	41 (20.2)	32 (25.2)	
**Body mass index (kg/m**^**2**^**)**				
<20	84 (25.5)	43 (21.2)	41 (32.3)	0.024^*^
≥20	246 (74.5)	160 (78.8)	86 (67.7)	
**Metastasis**				
No	285 (86.4)	179 (88.2)	106 (83.5)	0.225
Yes	45 (13.6)	24 (11.8)	21 (16.5)	
**Surgery**				
No	167 (50.6)	92 (45.3)	75 (59.1)	0.015^*^
Yes	163 (49.4)	111 (54.7)	52 (40.9)	
**New diagnosis**				
No	244 (73.9)	152 (74.9)	92 (72.4)	0.624
Yes	86 (26.1)	51 (25.1)	35 (27.6)	
**Cancer type**				
Head and neck cancer	86 (26.1)	53 (26.1)	33 (26)	0.52
Thoracic tumor	96 (29.1)	55 (27.1)	41 (32.3)	
Breast cancer	53 (16)	33 (16.3)	20 (15.7)	
Abdominal tumor	63 (19.1)	38 (18.7)	25 (19.7)	
Other cancer	32 (9.7)	24 (11.8)	8 (6.3)	

*The significance of difference between percentages was measured by the Pearson Chi Square test*.

* *Significant 0.01 < P ≤ 0.05*.

**Figure 1:**
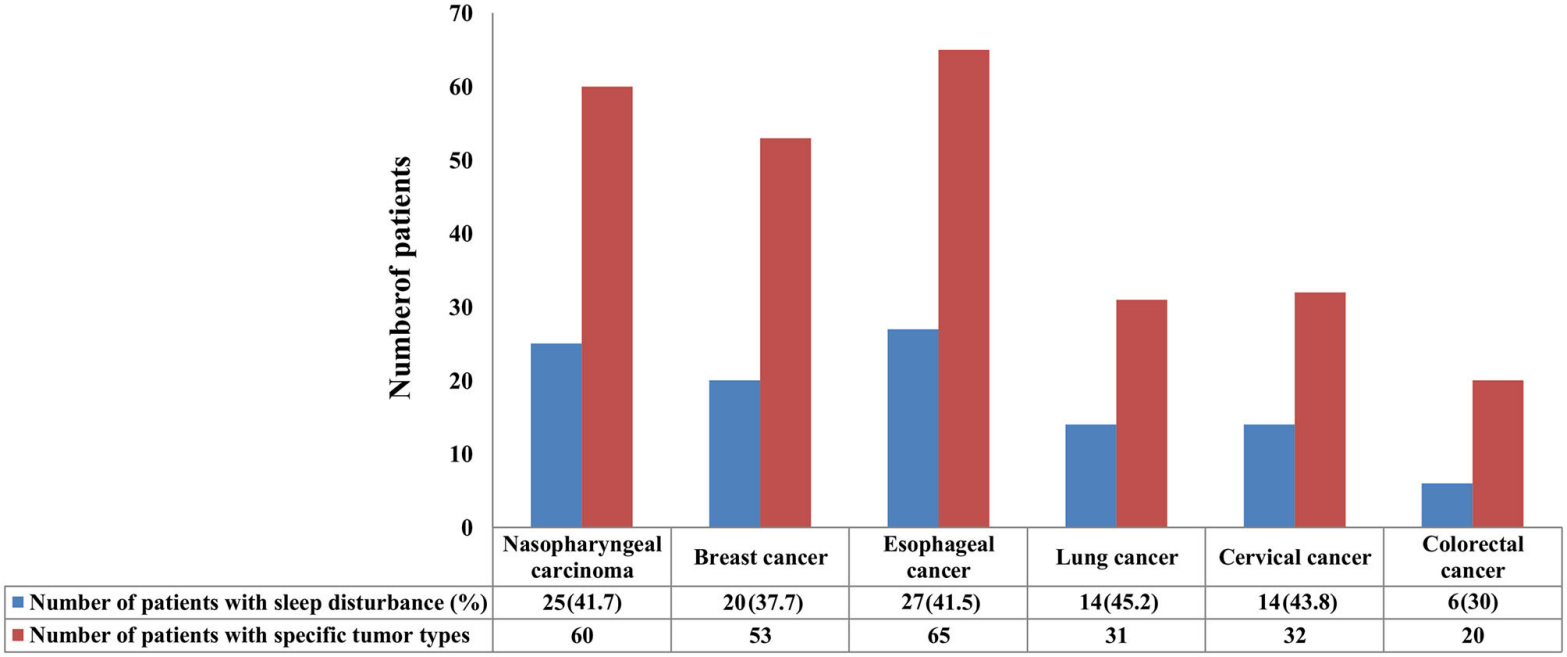
The incidence of sleep disturbance in patients with specific tumor types.

In this study, 41.7 (25/60), 37.7 (20/53), 41.5 (27/65), 45.2 (14/31), 43.8 (14/32), and 30% (6/20) of the patients with nasopharyngeal carcinoma, breast cancer, esophageal cancer, lung cancer, cervical cancer, and colorectal cancer suffered from sleep disturbance, respectively (Figure 1). In addition, patients with BMI <20 kg/m^2^ (*P* = 0.024) and non-surgery (*P* = 0.015) were more likely to suffer from sleep disturbance. However, there were no differences in gender, age, marital status, education level, comorbidity, metastasis status, diagnostic status, and cancer type (Table 1).

### Details of PSQI Score

The median global PSQI score was five (range: 0–17). Of the 330 patients, 38.3% (*n* = 127) had a global sleep quality score of more than seven, indicating that they suffered from a sleep disorder. One hundred and twelve patients (35.1%) needed more than 30 min to fall asleep, while 21.5% (*n* = 71) of them fell asleep in <15 min. The mean sleep duration was 8 h (range: 4–12 h), and 37.8 (*n* = 125), 27 (*n* = 89), 29.1 (*n* = 96), and 6.1% (*n* = 20) of them slept >7, 6–7, 5–6, and <5 h in a night, respectively. Regarding subjective sleep quality, 20.3% (*n* = 67) of the patients suffered fairly-very bad quality. In addition, among all the patients, 39.7% (*n* = 131) experienced sleep efficiency <85, and 6.1% (*n* = 20) needed medication to improve sleep, and 53.6% (*n* = 177) suffered day time dysfunction (having difficulties staying awake during the day time) at least once a week. Moreover, 78.2% (*n* = 258) of the patients had troubles sleeping at least once a week due to a variety of reasons such as shortness of breath, coughing or snoring, cold, hot, bad dreams, pain, and other reasons (Table 2).

**Table 2:** Descriptive statistics for the scores of the seven pittsburgh sleep quality index components in cancer patients.

**PSQI component score**	***N* (%)**	**Mean score (range)**	**IQR**
Subjective sleep quality		1.012 (0–3)	0
0 (very good)	72 (21.8)		
1 (fairly good)	191 (57.9)		
2 (fairly bad)	58 (17.6)		
3 (very bad)	9 (2.7)		
Sleep latency		1.245 (0–3)	1
0	58 (17.6)		
1	167 (50.6)		
2	71 (21.5)		
3	34 (10.3)		
Sleep duration		1.03 (0–3)	2
0 (> 7 h)	125 (37.8)		
1 (6–7 h)	89 (27)		
2 (5–6 h)	96 (29.1)		
3 (<5 h)	20 (6.1)		
Habitual sleep efficiency		0.706 (0–3)	1
0 (>85%)	199 (60.3)		
1 (75–85%)	68 (20.6)		
2 (65–75%)	24 (7.3)		
3 (<65%)	39 (11.8)		
Sleep disturbance		0.909 (0–3)	0
0	72 (21.8)		
1	218 (66.1)		
2	38 (11.5)		
3	2 (0.6)		
Use of sleep medication		0.109 (0–3)	0
0 (no use)	310 (93.9)		
1 (<1/week)	10 (3)		
2 (1–2/week)	4 (1.2)		
3 (≥3/week)	6 (1.9)		
Daytime dysfunction		0.906 (0–3)	2
0	153 (46.4)		
1	90 (27.3)		
2	52 (15.8)		
3	35 (10.5)		
Total score		5.92 (0–17)	5
≤ 7	203 (61.5)		
>7	127 (38.5)		

*IQR, interquartile range*.

### Predictors Associated With Sleep Disturbances

Multivariate logistic regression models showed that BMI ≥ 20 kg/m^2^ [odds ratio (OR) 0.599, 95% confidence interval (CI) 0.329–0.948, *P* = 0.031] and receipt of surgery (OR 0.507, 95% CI 0.258–0.996, *P* = 0.048) were significant favorable predictors for sleep disturbance (Table 3). However, gender (*P* = 0.089), age (*P* = 0.491), marital status (*P* = 0.498), education level (*P* = 0.205), comorbidity (*P* = 0.342), metastasis status (*P* = 0.794), diagnostic status (*P* = 0.489), and cancer type (*P* = 0.882) were not associated with sleep disturbance.

**Table 3:** Predictors of sleep disturbance in cancer patients.

**Variables**	**OR**	**95% CI**	***P***
**Gender**				
Male	1		
Female	1.61	0.929–2.790	0.089
**Age (years)**				
<40	1		0.491
40–60	1.606	0.734–3.510	0.235
>60	1.461	0.615–3.472	0.39
**Marital status**				
Married	1		
Unmarried or divorced	0.763	0.350–1.666	0.498
**Education level**				
Middle school or below	1		
High school or above	1.503	0.800–2.824	0.205
**Comorbidity**				
No	1		
Yes	1.318	0.746–2.329	0.342
**Body mass index (kg/m**^**2**^**)**				
<20	1		
≥20	0.559	0.329–0.948	0.031^*^
**Metastasis**				
No	1		
Yes	1.101	0.535–2.266	0.794
**Surgery**				
No	1		
Yes	0.507	0.258–0.996	0.048^*^
**New diagnosis**				
No	1		
Yes	0.77	0.367–1.616	0.489
**Cancer type**				
Head and neck cancer	1		
Thoracic tumor	0.963	0.472–1.965	0.918
Breast cancer	0.998	0.389–2.563	0.997
Abdominal tumor	0.906	0.400–2.055	0.814
Other cancer	0.599	0.210–1.712	0.339

*OR, odds ratio; CI, confidence interval*.

*The significance of difference between percentages was measured by the multivariate logistic regression analysis*.

* *Significant 0.01 < P ≤ 0.05*.

When stratifying according to surgery, BMI ≥ 20 kg/m^2^ (OR 0.287, 95% CI 0.119–0.691, *P* = 0.005) was still a favorable predictor for sleep quality in patients receiving surgery, while the remaining factors had no significance. However, in patients not receiving surgery, female (OR 2.656, 95% CI 1.264–5.581, *P* = 0.01) was an adverse factor, while the other factors had no association with sleep disorders (Table 4).

**Table 4:** Significant predictors of sleep disturbance stratified by surgery in patients with cancer.

**Variables**	**Surgery**			**Non-surgery**		
	**OR**	**95%CI**	***P***	**OR**	**95%CI**	***P***
**Gender**						
Male	1			1		
Female	0.635	0.236–1.714	0.37	2.656	1.264–5.581	0.01^**^
**Age (years)**						
<40	1			1		
40–60	1.837	0.551–6.127	0.322	2.088	0.648–6.726	0.217
>60	2.649	0.635–11.042	0.181	1.541	0.440–5.398	0.499
**Marital status**						
Married	1			1		
Unmarried or divorced	0.429	0.109–1.690	0.226	3.142	0.844–11.702	0.088
**Education level**						
Middle school or below	1			1		
High school or above	1.836	0.664–5.078	0.241	1.48	0.585–3.746	0.407
**Comorbidity**						
No	1			1		
Yes	0.556	0.199–1.556	0.264	2.047	0.924–4.534	0.077
**Body mass index (kg/m**^**2**^**)**						
<20	1			1		
≥20	0.287	0.119–0.691	0.005^**^	0.701	0.339–1.448	0.337
**Metastasis**						
No	1			1		
Yes	0.71	0.124–4.083	0.701	0.942	0.402–2.205	0.891
**New diagnosis**						
No	0			1		
Yes	—	—	—	0.623	0.269–1.444	0.27
**Cancer type**						
Head and neck cancer	1			1		
Thoracic tumor	2.626	0.528–13.066	0.238	0.739	0.297–1.837	0.515
Breast cancer	4.543	0.873–23.642	0.072	–	–	–
Abdominal tumor	4.324	0.852–21.941	0.077	0.325	0.103–1.020	0.054
Other cancer	1.268	0.249–6.459	0.775	0.371	0.047–2.944	0.348

*OR, odds ratio; CI, confidence interval*.

*The significance of difference between percentages was measured by the multivariate logistic regression analysis*.

** *Significant P ≤ 0.01*.

## Discussion

In the present study, we aimed to investigate the prevalence of sleep disturbance and the risk factors associated with the development of sleep disorders among patients with cancers. Our study found that ~40% of cancer patients suffered from sleep disturbance. With regard to the PSQI component scores, a high proportion of patients reported poor sleep quality, poor sleep efficiency, and day time dysfunction. In addition, BMI ≥ 20 kg/m^2^ and the receipt of surgery were the significant favorable predictors for sleep disorders.

The prevalence of sleep disturbance was frequently discussed in patients with cancers, while less attention was paid to the sleep problems in the context of radiotherapy. In this study, 38.3% of the cancer patients had sleep disorders before the start of radiotherapy, which was consistent with the findings from previous studies (30–50%), and this result extends the findings of sleep disturbance in the whole process management of cancer patients (20, 21). In addition, prior studies had reported that insomnia was the most common aspect of sleep disturbance when measuring sleep quality before or after chemotherapy (7, 8, 21). A similar result was found in our study that ~30% of the patients reported varying degrees of insomnia before radiotherapy, including poor sleep efficiency, longer sleep latency, or shorter sleep duration. It is noteworthy that various studies had demonstrated that sleep problems had a significant impact on psychological well-being and QOL among cancer patients (21–23). Therefore, this negative effect must be taken into consideration throughout the treatment, even when radiotherapy was administered.

Most studies focused on sleep problems in patients with cancer, and the probabilities of sleep disturbance in different tumor subtypes showed significant discrepancies (19, 23–26). As reported in previous studies, sleep disturbance was focused on patients with breast cancer, and the incidence of sleep problems ranged from 39.5 to 69% (19, 23). However, other studies showed that patients with lung cancer had the poorest sleep quality compared with other cancer types or control populations (24, 25). A similar result was found in the cureent study that patients with lung cancer had the highest proportion of sleep disorders (45.2%) before radiotherapy compared with other cancer types. The possible explanation was that patients with lung cancer were more likely to suffer from hypoxia and disordered breathing associated with the primary lesion and physiologic changes, leading to insomnia, falling asleep difficultly, and intermittent sleep (26, 27). Hence, more attention should be paid to patients with lung cancer for their sleep problems to improve the QOL.

We further explored the independent risk factors associated with sleep disturbance, and the result showed that BMI ≥ 20 kg/m^2^ was one of the significant favorable predictors. The result was inconsistent with the finding from Bardwell et al. which showed that BMI had no association with sleep disorders (28). It is noteworthy that the evaluation instrument in their study was different from that in our study, and scores of more than nine were defined as poor sleep quality. Moreover, the participants in their study only covered breast cancer patients (28), while all cancer types were included in our study, which might be the reason for the difference. Conversely, another study from the United States included 73 patients with cancer during or after radiotherapy, and the result showed that higher BMI was associated with poor sleep quality (29). It should be noted that the cut-off value of BMI in their study was 27.4 (the average BMI), and 30% had a BMI of more than 30 kg/m^2^. To the best of our knowledge, obesity has a significantly close association with sleep problems; hence the cut-off value of 27.4 might not accurately reflect the relationship between BMI and sleep quality in cancer patients (29–31). In this study, BMI ≥ 20 kg/m^2^ was a protective factor for sleep disturbance, and only 1.8% of the patients had a BMI of more than 30. These results might surmise that a mild overweight was beneficial to sleep quality in cancer patients.

Our results confirm that surgery was another favorable predictor for sleep disorders in cancer patients. The result was consistent with the finding from a review in 2019 (16). The reason why surgery has a positive effect on sleep quality might be that patients receiving surgery have adapted themselves to the role of cancer patients and the environment of the hospital before the start of radiotherapy, and less anxiety and depression could lead to fewer sleep problem (32). In this study, 31.9% of the patients treated with surgery suffered from sleep disturbance, while 44.9% of the patients who were not receiving surgery had poor sleep quality. In addition, stratification analysis showed that female was a significantly adverse predictor for sleep disturbance in patients not receiving surgery. The possible explanation was that distinct hormonal and physical differences made women more vulnerable to diseases, especially to cancer, and sleep disturbance became one of the most common presentations (33). Therefore, more attention should be paid to patients who were not receiving surgery, especially in female patients, and medication aid could be given if necessary.

Several limitations should be acknowledged in this study. Firstly, all types of cancer were included in our institution, so that the sample size of each tumor site was small. Secondly, the subjective sleep quality was investigated in this study, while objective sleep quality was not. Thirdly, only PQSI scale was used in our study, additional information regarding sleep quality, such as the Epworth sleepiness scale and the evaluation of the psychological profile of cancer patients, should be incorporated to verify our findings. Moreover, our study lacks the evaluation of associated psychiatric comorbidity such as anxiety, depression, and day time sleepiness, which might affect the results. Finally, previous studies had shown that patients with head and neck cancer who received radiotherapy were more likely to develop sleep disturbance (34). However, our study only assessed the risk of sleep disturbances before radiotherapy in our department. In the future, we will further study the changes of sleep quality in cancer patients during radiotherapy.

## Conclusion

In conclusion, our study suggests that ~40% of cancer patients suffer from sleep disturbance before the start of radiotherapy. Patients with BMI ≥ 20 kg/m^2^ and receiving surgery were significant favorable predictors for sleep quality. More clinical studies should be conducted to explore sleep disturbance in cancer patients in the context of radiotherapy.

## Data Availability Statement

The raw data supporting the conclusions of this article will be made available by the authors, without undue reservation.

## Ethics Statement

The studies involving human participants were reviewed and approved by the First Affiliated Hospital of Xiamen University. The patients/participants provided their written informed consent to participate in this study.

## Author Contributions

H-JL and S-GW contributed to the conception, designed the study, and took full responsibility for the whole work. PZ, JW, and C-LL performed analyses. JW and B-YZ analyzed results and wrote the manuscript. All authors contributed to the article and approved the submitted version.

## Conflict of Interest

The authors declare that the research was conducted in the absence of any commercial or financial relationships that could be construed as a potential conflict of interest.
